# First complete mitochondrial genome of *Phoenix dact ylifera* var. Khanezi

**DOI:** 10.1080/23802359.2018.1491339

**Published:** 2018-07-11

**Authors:** Sajjad Asaf, Abdul Latif Khan, Ahmed Al-Harrasi, Ahmed Al-Rawahi

**Affiliations:** Natural and Medical Science Research Center, University of Nizwa, Nizwa, Oman

**Keywords:** Mitochondrial genome, *Phoenix dactylifera*, Arecaceae, phylogenetic analysis, sequencing

## Abstract

In this study, we determined the complete mitochondrial (mt) genome of *Phoenix dactylifera* var. Khanezi. The results revealed a circular genome of 715,120 bp, having G + C content of 45.1%, containing 40 protein coding genes, 3 rRNA, and 18 rRNA genes. Evolutionary relationship analysis suggested that *P. dactylifera* var. Khanezi is more closely related to previously reported *P. dactylifera* var. Khalas.

Date palm, *Phoenix dactylifera* L., belongs to *Arecaceae* and is an ecologically, culturally and economically important fruit tree in North Africa and Middle East (Moussouni et al. 2017). Date palm is a perennial, monocotyledon (2*n* = 36), dioecious, cross-pollinated tree that has been widely cultivated (Barrow 1998; Terral et al. 2012; Cherif et al. 2013). Morphological variations, which are heavily dependent on environmental factors and data variety, do exist among cultivars. These variations are reflected in the diversity of the chloroplast (cp) genome, and mt genomics as well.

The mitochondria play a vital role in plant growth and development (Ogihara et al. 2005). Recently, mt genomic gained much attention due to advancement in genomics, and mt DNA is considered as a significant and effective source of genetic variation among various species. The mt genomes of plant have big and complicated structure as compared to other eukaryotes (Li et al. 2009; Liu et al. 2011). Furthermore, angiosperm mt genomes are well known for uptake of foreign DNA by horizontal gene transfer (Goremykin et al. 2009) and for their very low mutation rate (Palmer and Herbon 1988). In the present study, we report for the first time the complete nucleotide sequence of *P. dactylifera* var. Khanezi mt genome (GenBank accession number: NC016740) and to infer its phylogenetic position on the basis of entire mito-genomes.

The *P. dactylifera* var. Khanezi (accession), plants were received from the GenBank at the University of Nizwa, Nizwa Oman. Young leaves were used to extract mitochondrial DNA by using the method described previously (Asaf et al. 2016; Wu 2016) with little modification. The living material and DNA were stored in University of Nizwa, Nizwa, Oman. Geneious Pro v11.1 (http://geneious.com) was used to filter and assemble the raw alumina reads. All contigs were blasted using the NCBI database (http://www.ncbi.nlm.nih.gov/) for the annotation of mitochondrial sequences. Similarly, tRNA scan-SE software (http://lowelab.ucsc.edu/tRNAscan-SE/) was used to identify tRNAs. The entire mitochondrial genome was used to determine its phylogenetic position using Maximum persimony tree with 1000 bootstrap replications (Felsenstein 1985).

The mitochondrial genome of *P. dactylifera* var. Khanezi was assembled as a single circular molecule of 715,120 bp having 45.1% GC contents (deposited in GenBank under the accession MH176159). The largest part of the *P. dactylifera* mtDNA comprises the non-coding sequences (93.2%), which is slightly larger than the average non-coding sequences content (89.45%) in other angiosperm mt genomes (Chaw et al. 2008). Furthermore, the mt genome contains 67 genes encoding 24 transfer RNAs, 3 ribosomal RNAs and 40 protein coding genes. Moreover, the phylogenetic analysis revealed that mt genome of *P. dactelifera* var. Khanezi is closely related to *P. dactelifere* var. Khalas (NC016740; Figure 1) and *Cocos nucifera*. This study will help to understand the evolution of various date palm cultivars mitochondrial genome with related species.

**Figure 1.**
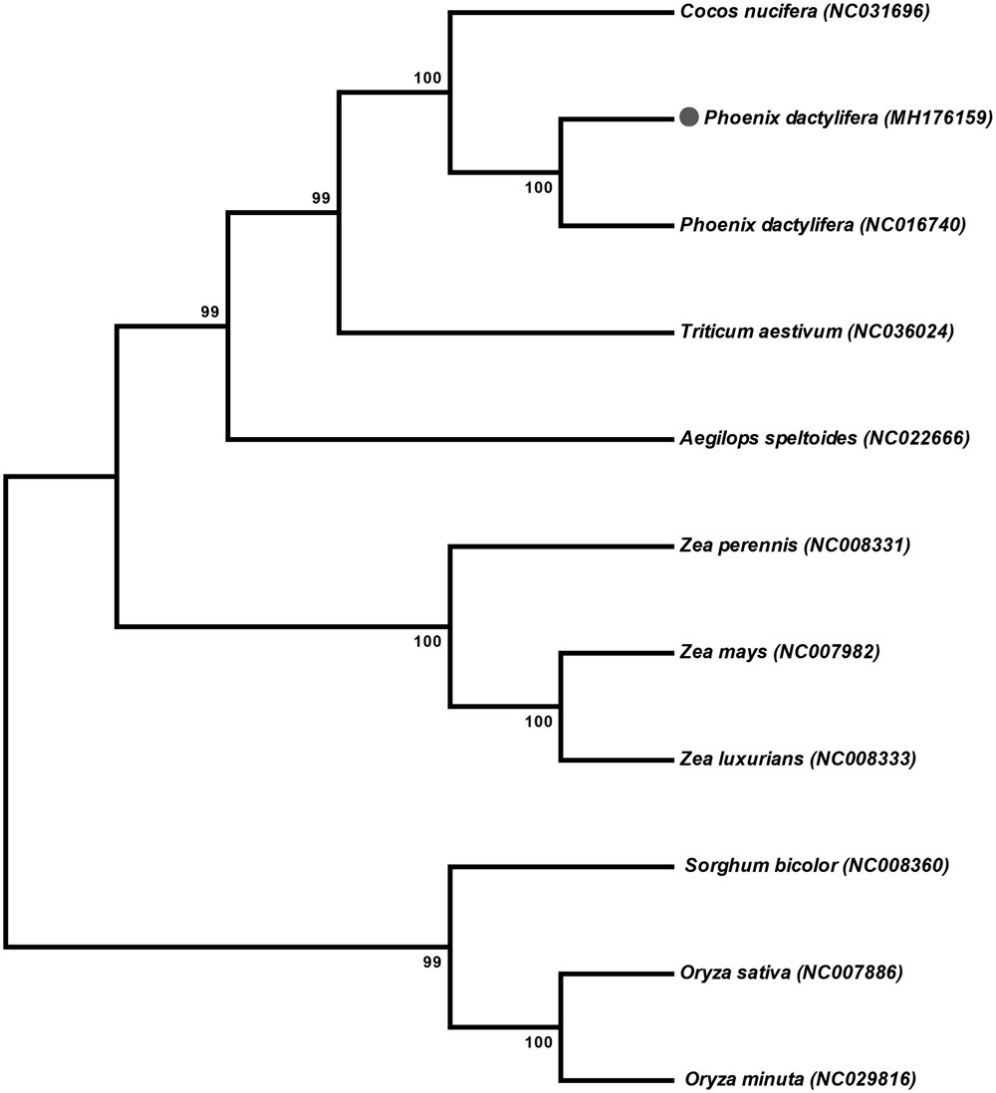
Phylogeny of the *Phoenix dactylifera* var. Khanezi mitochondrial genome with ten other related species (two from family Arecaceae and eight from family Poaceae). The phylogenetic tree was inferred using the Maximum parsimony method based on entire mitochondrial genomes of these species.

## References

[CIT0001] Asaf S, Khan AL, Khan AR, Waqas M, Kang S-M, Khan MA, Shahzad R, Seo C-W, Shin J-H, Lee I-J 2016 Mitochondrial genome analysis of wild rice (*Oryza minuta*) and its comparison with other related species. PloS One. 11:e0152937. 27045847 10.1371/journal.pone.0152937 PMC4821559

[CIT0002] Barrow SC 1998 A monograph of *Phoenix* L. (Palmae: Coryphoideae). Kew Bull. 53:513–575.

[CIT0003] Chaw S-M, Chun-Chieh Shih A, Wang D, Wu Y-W, Liu S-M, Chou T-Y 2008 The mitochondrial genome of the gymnosperm Cycas taitungensis contains a novel family of short interspersed elements, Bpu sequences, and abundant RNA editing sites. Mol Biol Evol. 25:603–615. 18192697 10.1093/molbev/msn009

[CIT0004] Cherif E, Zehdi S, Castillo K, Chabrillange N, Abdoulkader S, Pintaud JC, Santoni S, Salhi-Hannachi A, Glémin S, Aberlenc-Bertossi F 2013 Male‐specific DNA markers provide genetic evidence of an XY chromosome system, a recombination arrest and allow the tracing of paternal lineages in date palm. New Phytol. 197:409–415. 23231423 10.1111/nph.12069

[CIT0005] Felsenstein J 1985 Confidence limits on phylogenies: an approach using the bootstrap. Evolution. 39:783–791. 28561359 10.1111/j.1558-5646.1985.tb00420.x

[CIT0006] Goremykin VV, Salamini F, Velasco R, Viola R 2009 Mitochondrial DNA of Vitis vinifera and the issue of rampant horizontal gene transfer. Mol Biol Evol. 26:99–110. 18922764 10.1093/molbev/msn226

[CIT0007] Li L, Wang B, Liu Y, Qiu Y-L 2009 The complete mitochondrial genome sequence of the hornwort Megaceros aenigmaticus shows a mixed mode of conservative yet dynamic evolution in early land plant mitochondrial genomes. J Mol Evol. 68:665–678. 19475442 10.1007/s00239-009-9240-7

[CIT0008] Liu Y, Xue J-Y, Wang B, Li L, Qiu Y-L 2011 The mitochondrial genomes of the early land plants Treubia lacunosa and Anomodon rugelii: dynamic and conservative evolution. PLoS One. 6:e25836. 21998706 10.1371/journal.pone.0025836 PMC3187804

[CIT0009] Moussouni S, Pintaud JC, Vigouroux Y, Bouguedoura N 2017 Diversity of Algerian oases date palm (Phoenix dactylifera L., Arecaceae): Heterozygote excess and cryptic structure suggest farmer management had a major impact on diversity. PLoS One. 12:e0175232. 28410422 10.1371/journal.pone.0175232 PMC5391916

[CIT0010] Ogihara Y, Yamazaki Y, Murai K, Kanno A, Terachi T, Shiina T, Miyashita N, Nasuda S, Nakamura C, Mori N 2005 Structural dynamics of cereal mitochondrial genomes as revealed by complete nucleotide sequencing of the wheat mitochondrial genome. Nucleic Acids Res. 33:6235–6250. 16260473 10.1093/nar/gki925 PMC1275586

[CIT0011] Palmer JD, Herbon LA 1988 Plant mitochondrial DNA evolves rapidly in structure, but slowly in sequence. J Mol Evol. 28:87–97. 3148746 10.1007/BF02143500

[CIT0012] Terral JF, Newton C, Ivorra S, Gros-Balthazard M, de Morais CT, Picq S, Tengberg M, Pintaud JC 2012 Insights into the historical biogeography of the date palm (Phoenix dactylifera L.) using geometric morphometry of modern and ancient seeds. Journal of Biogeography. 39:929–941.

[CIT0013] Wu Z 2016 The whole chloroplast genome of shrub willows (Salix suchowensis). Mitochondr DNA A. 27:1–2154. 10.3109/19401736.2014.982602 25418623

